# Text Messaging for Mental Health Promotion With Migrants Returning to Mexico: Content Development and Piloting With a Needs Assessment Approach

**DOI:** 10.2196/83365

**Published:** 2026-05-26

**Authors:** Marcela Tiburcio, Sarahí Alanís Navarro, Shoshana Berenzon, Raquel Mondragón Gómez, María Elena Rivera Heredia

**Affiliations:** 1 Departamento de Ciencias Sociales en Salud, Dirección de Investigaciones Epidemiológicas y Psicosociales, Instituto Nacional de Psiquiatría Ramón de la Fuente Muñiz, Mexico City, Mexico; 2 Dirección de Investigaciones Epidemiológicas y Psicosociales, Instituto Nacional de Psiquiatría Ramón de la Fuente Muñiz, Calzada México-Xochimilco 101, San Lorenzo Huipulco, Mexico City, 14370, Mexico, 52 5541605168; 3 Seminario de Estudios sobre la Globalidad: Migración, Facultad de Medicina, Universidad Nacional Autónoma de México, Mexico City, Mexico; 4 Facultad de Psicología, Universidad Nacional Autónoma de México, Mexico City, Mexico; 5 Facultad de Psicología, Universidad Michoacana de San Nicolás de Hidalgo, Morelia, Michoacán, Mexico

**Keywords:** migrants, mental health, substance use, digital intervention, WhatsApp

## Abstract

**Background:**

Returning migrants face a variety of challenges that limit their ability to integrate and adapt to Mexico. This represents a break in their life trajectory, with effects on family dynamics, mid- and long-term projects, and uncertainty about short-term plans.

**Objective:**

This study describes the coproduction approach used to design and develop a WhatsApp-based psychoeducational program entitled “Here Again: Coping With Return,” which aims to promote the adoption of self-care behaviors to reduce the risk of mental health and substance use problems among returning migrants.

**Methods:**

The process included four phases: (1) a situational diagnosis of the needs of migrants in preventing mental health problems and reducing the risks associated with alcohol use, (2) the design and development of content, (3) evaluation by a group of experts in mental health and substance use, and (4) pilot testing.

**Results:**

The study identified 4 intervention pillars: emotional risk factors, coping strategies, barriers to care, and technological feasibility. Eighty WhatsApp messages were developed, focusing on mental health (n=52, 65%) and alcohol use (n=20, 25%) through a sequence of motivation, instruction, and reinforcement. Following an expert evaluation that simplified technical language, a pilot study with 14 migrants showed a 78.6% completion rate. Participants reported the successful application of emotional management tools and a preference for text-based messages over audiovisual content to conserve mobile data.

**Conclusions:**

This study describes the development of a psychoeducational program for returning migrants based on coproduction, integrating user needs and expert experience. The intervention addresses emotional management, self-care, and substance use prevention, using WhatsApp for its accessibility and low cost. The pilot results demonstrated high acceptability and a 78.6% retention rate over 16 weeks, highlighting that the culturally sensitive approach and accessible language enabled participants to apply mental health tools autonomously and effectively.

## Introduction

Recent years have seen significant changes in the profiles of migrants to the United States who return to Mexico. The returning migrants show an increase in the number of people returning alone, including a greater number of women, although there are still more men who return alone (76% men vs 23% women). The age at return has also decreased, with a substantial number returning at working age. The majority have lived in the United States for more than 10 years, and they have spent more than half of their lives there [1,2].

Returning migrants face a variety of challenges that limit their ability to integrate and adapt to Mexico. Many leave the United States suddenly after being deported or choosing “voluntary departure” because they do not have the necessary documentation to remain in the country. Even those who have time to prepare may not be able to find the required information [3]. Therefore, returning to Mexico can mean a break in their life trajectory, with effects on family dynamics, mid- and long-term projects, and uncertainty about short-term plans [4].

Upon arriving in Mexico, they encounter a cumbersome bureaucracy, limited access to labor and educational markets, and social security, and their living conditions are often worse than before they migrated. All these conditions, on top of social stigma and feelings of failure, impotence, and frustration, contribute to the development of mental health problems [3]. Returning migrants frequently have insufficient access to health care, the result of legal, political, and financial limitations. Even when they have formal access, they have difficulty using these services owing to cultural barriers, stigma, and structural obstacles [5].

One option for reducing the risk of such problems is psychoeducation, and for people like returning migrants who are difficult to reach, digital tools are an effective medium for promoting change. Interventions using smartphones are widely recognized as useful tools for the promotion of healthy habits, self-care behaviors, and harm reduction [6-8].

Various authors have noted that participatory methods of “coproduction” are ideal for assuring that users’ needs are satisfied and for promoting long-term commitment. Coproduction methods are characterized by the joint participation of researchers, professionals, and the public, working together, respecting, and valuing a variety of knowledge, as well as sharing power and responsibility from the beginning to the end of the project [9]. This collaborative approach can help to identify perspectives and ideas in addition to those of the research team [10].

This study describes the coproduction approach to designing and developing a WhatsApp-based psychoeducational program entitled “Here Again: Coping With Return,” which aims to promote the adoption of self-care behaviors to reduce the risk of mental health problems in returning migrants. The WhatsApp messages contain information about self-care practices and strategies for harm reduction and reducing risks associated with alcohol use. Throughout the development process, researchers, software developers, returning migrants, and people who provide direct care to this population worked together to ensure that the program was sensitive to the needs of people who return to Mexico.

## Methods

**Figure 1.**
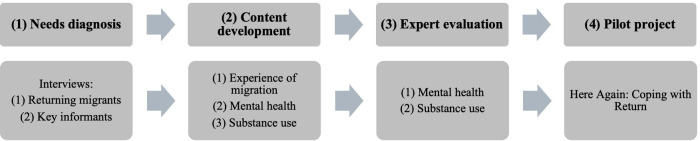
General description of the program development process.

The development of the “Here Again: Coping With Return” program included four phases: (1) a situational diagnosis of the needs of migrants in preventing mental health problems and reducing the risks associated with alcohol use, (2) the design and development of content, (3) evaluation by a group of experts in mental health and substance use, and (4) the development and execution of a pilot study (Figure 1).

### Ethical Considerations

The research was carried out according to the guidelines of the Ethics Committee of the Ramón de la Fuente National Institute of Psychiatry, which approved the research protocol (approval number CEI/C/023/2021) [11]. Participation was free and voluntary. Pseudonyms chosen by the participants were used to maintain confidentiality. Those who experienced more complex mental health conditions and needed more intensive help could send the word “support” to the platform administration to be referred to a psychiatrist who would respond within 24 hours.

### Phase 1: Needs Diagnosis

To ensure that the program was sensitive to the needs of migrants returning to Mexico, this phase explored the difficulties they faced with respect to mental health care and substance use, as well as their perception of the usefulness of a message-based psychoeducational program.

#### Design

The study design was qualitative, using semistructured interviews with returning migrants and key informants. The interview topics were established beforehand, but they included a degree of flexibility while maintaining sufficient uniformity to facilitate interpretation in accordance with the purposes of the study [12].

#### Locations

The interviews were carried out in the cities of Tijuana, a city on the border with the United States, and Morelia, the capital of Michoacán, a state with a long history of migration that also has one of the largest populations of people repatriated to their places of origin. These locations were included to obtain the perspectives of people just returning to Mexico and of those who have arrived in their communities of origin or other destinations.

#### Participants

To capture the multidimensionality and richness of return experiences, 2 heterogeneous samples were selected to allow for a comparison of diverse realities within the migration process.

- *Returning migrants*: These included Mexican men and women aged 18 years and older who had migrated to the United States and returned to Mexico within the previous 24 months.
- *Key informants*: These included people with experience, knowledge, or a role within an institution or organization that made them an appropriate source of information.

The broadness of the inclusion criteria ensured that the analysis relied not only on official data but also on the direct experiences of those who experience, manage, support, or investigate the social, legal, and human complexities that migrants face upon their return.

#### Instruments

A thematic interview guide was used, exploring (1) sociodemographic data, including time in the United States; for returning migrants, the time elapsed between return and the interview; for key informants, the length of time they had worked with migrants; (2) experience of migration, including conditions of return and difficulties during the adaptation process; (3) emotional difficulties, substance use, and use of health services; and (4) perceptions of the usefulness of a SMS text messaging–based psychoeducational strategy.

#### Procedure

Participants were contacted through a snowball sampling method and organizational connections. The interviews were carried out face-to-face and lasted an average of 1 hour. Participants were given information about the objective of the study, the characteristics and length of the interview, and the confidentiality of the data. They provided informed consent to participate in the study and for the interview to be recorded. The returning migrants were given a gift card for groceries in recognition of their time and effort, and informational activities and printed materials on mental health topics were provided to the key informants’ organizations.

#### Data Analysis

The data were analyzed using the meaning categorization technique [13], which facilitates the extraction of concise discursive units for systematic comparison. This technique aligns with an interpretive epistemology that places people’s subjectivity at the center of analysis.

Following multiple readings of the interview transcripts to identify key themes for the needs assessment, 2 researchers independently coded the data. Interrater discrepancies were resolved through iterative discussion and a joint review of the original transcripts. The resulting analytical categories included risk factors, resources and coping strategies, barriers to help-seeking, and the feasibility of a message-based intervention.

### Phase 2: Design and Development of Content

#### Overview

**Figure 2.**
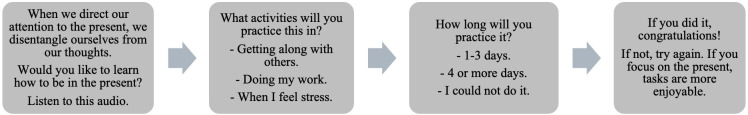
Example of the sequence of messages about adopting a practice of mindfulness.

Based on the diagnosis, psychoeducational messages were prepared on three main topics:

1. *Experience of migration*: It addresses the risk and protective factors associated with emotional problems and alcohol use in the migrant population. The information is presented at the beginning of each topic with a video in which a couple describes their experience of migration and shares the potential benefits of participating in the program. This resource seeks to motivate participants to stay in the program, based on self-identification. The videos were produced and performed by members of the participant communities.
2. *Mental health*: It includes 2 subtopics: management of emotions and self-care behaviors. The messages regarding emotional management are divided into 2 parts. The first part describes the characteristics of the 3 emotions most frequently mentioned by the interviewees during the needs detection phase: sadness, anxiety, and anger. It responds specifically to 3 questions: “What is the function of the emotion?” “What does it feel like?” and “What can I do when I feel it?” The second part presents mindfulness-based emotion regulation strategies, as outlined in the guide “Doing What Matters in Times of Stress” [14]. The literature indicates that these strategies are particularly suitable for brief, scalable, and low-cost interventions and that they have demonstrated positive effects in contexts of adversity [15]. Figure 2 illustrates the sequence of messages for incorporating emotional regulation exercises. In addition to integrating the ideas and opinions of the migrants and key informants, the preparation of the messages considered the concept of the “Big Five” [16], 5 groups of activities associated with mental health: healthy thoughts, meaningful activities, plans and goals, healthy routines (meals, sleep, and exercise), and social connection. The messages highlight the benefits of activity and promote commitment to action, encouraging participants to choose a realistic goal and monitor their involvement in change, and they provide recognition of success and a reminder of the reasons for adopting the habit. At the end of the section, a message describes the indicators that suggest a mental health problem and encourages the participant to seek help, with information about available local options.
3. *Alcohol use*: It includes strategies to promote low-risk use, some of which are based on the Drink Less program [17]. These include the identification of situations of risk for use, such as negative emotional states, awareness of behaviors incompatible with making changes, the risks of excessive use, information on the limits of low-risk use, help in establishing goals to reduce risks and harms, and alternatives for attaining them. The information about maintaining low-risk use levels includes recommendations for lowering the amount of alcohol and other substances and a directory of local services to facilitate seeking help.

#### Procedure

The development of the messages was carried out using a conceptual matrix that described the topic, subtopic, objective, content, and the scope and format of the message (Textbox 1). The scope of the messages was based on the recommendation of Walters [18] to shape behaviors using motivation, ability, and triggers. Motivation was promoted with an emphasis on the participants’ self-identification, providing information about the benefits of change and recognition of success. Ability consisted of guidelines to facilitate behavior, based on the modeling of activities, setting realistic goals, and reducing obstacles. Triggers provided alternatives for situations and moments in which behaviors were most likely, with questions about the level of involvement and reminders of goals.

Textbox 1. **Example of the conceptual matrix for the development of messages.**

Mental health and management of emotions: provide information about the functions of the emotions and strategies for managing them

- Function of sadness: What is sadness?
  - Message
    - We feel sadness when we lose something valuable, such as a relationship, a loved one, a job, our health, a project, or material possessions.
    - When we allow ourselves to feel sadness, we can reflect on what is happening to us and face change.
    - Sadness allows us to accept loss and move our lives in a new direction.
  - Scope
    - Inform motivation
  - Format
    - Image

- Practice being in the present
  - Message
    - This week, when will you practice the exercise for being in the present?
      - In getting along with others
      - In doing my job
      - When I feel stressed
  - Scope
    - Trigger
  - Format
    - Questionnaire

### Phase 3: Expert Evaluation

The purpose of the third phase was to evaluate the usefulness of the information in achieving the objectives of the project.

#### Design

Validation of the project was carried out through an expert evaluation by experts in mental health and alcohol use, following an overlapping design [19,20].

#### Participants

Seven researchers participated in the evaluation, comprising both men and women, 3 of them with experience in the field of substance abuse and 4 in the field of mental health. Each participant evaluated an average of 30 messages. To provide a balance, the mental health topics were divided into 2 subtopics, management of emotions and self-care, and included introductory messages.

Expert evaluation is a method for verifying the reliability of a study based on the informed opinion of persons recognized as qualified experts in the subject matter. The experts contribute information, evidence, judgments, and evaluations [21].

#### Instrument

A questionnaire was used to evaluate the content of the messages, with responses rated on a 5-point Likert scale (1=unsuitable to 5=well-suited). Evaluators assessed the messages based on several criteria: agreement of the message with the general topic, alignment with the objective, understandability, relevance, and appropriateness of the format. The criteria were framed as questions: (1) How well does the content of the message relate to the topic? (2) How well does the message contribute to achieving the goal of the section? (3) How understandable is the language? (4) How relevant is the information in the message? and (5) How appropriate is the format of the message? Evaluators could also include their own commentaries.

The questionnaire concluded with a final section to evaluate the entire set of messages on a given topic. This section comprised five questions: (1) How appropriate is the topic for the purposes of the project? (2) Is the number of messages in this section appropriate? (3) How practical are the recommended exercises? (4) How appropriate are the messages for the target group? and (5) How encouraging are the messages? It also included a section for general comments.

#### Procedure

An invitation was sent to the experts’ institutional email describing the objective of the study, the tasks they would perform, and the ethical considerations. Those who accepted were sent an evaluation form and instructions. Each participant evaluated the messages related to their field of study, as well as the introductory messages.

#### Data Analysis

The average score was calculated for each criterion by question and by section. The maximum score was 5, and the minimum score was 1.

### Phase 4: Pilot Study of the Program “Here Again: Coping With Return”

The program “Here Again: Coping With Return” included 80 messages that responded to the needs identified in the situational diagnosis for coping with the emotional problems associated with return. A pilot study was conducted with the objective of evaluating whether the messages conveyed the expected information and adjusting the functioning of the platform and the general characteristics of sending and receiving messages.

#### Participants

Returning migrants with characteristics similar to those who would be included in the feasibility study were invited to participate in the pilot study. The inclusion criteria were being 18 years or older, having returned to Mexico within the past 24 months, using WhatsApp, and having the ability to understand and read Spanish. Participants were contacted through nongovernmental organizations and research groups that offer services to the migrant population in Ciudad Juárez, Chihuahua, and different municipalities of Michoacán.

#### Instruments

A questionnaire was administered before and after the intervention with the following sections:

- *Sociodemographic data and general state of health*: Participants were asked for such data as age, sex, birthplace, and religion. One question evaluated the perception of their state of health on a 5-point Likert scale from “very bad” to “very good.” Three other questions focused on health problems and the use of health services during the period of return.
- *Patient Health Questionnaire-4*: The 4-item Patient Health Questionnaire-4 [22] includes 4 questions that explore the frequency of symptoms of anxiety and depression in the previous 2 weeks. The response options are presented as a 4-point Likert scale from “never” to “almost every day.” The score indicates a level of psychological problems that can be normal, mild, moderate, or severe. It also includes subscales with cutoffs that suggested possible anxiety or depression. The Spanish version has been validated [23] and used with various migrant populations in Mexico [24,25].
- *The Alcohol Use Disorders Identification Test* [26]: This test consists of 10 questions with response options from 0 to 4 points; the sum indicates the risk level associated with alcohol use in the previous year: low, risky, harmful, or high. The questions are grouped into 3 dimensions: alcohol use, consequences of use, and symptoms of dependency. The cutoff for risky use is 8 [27].
- *Use of psychoactive substances*: The first 2 questions were administered from the Alcohol, Smoking, and Substance Involvement Screening Test [28]. These explore lifetime use and use in the previous 3 months of 8 substances: tobacco, cannabis, cocaine, amphetamines, inhalants, sedatives, hallucinogens, and opioids. It has been validated for the Mexican population [29].
- *Opinions about the program*: An ad hoc questionnaire was administered at the follow-up interview with 8 closed and 8 open-ended questions about participants’ experiences in the program, the usefulness of the information, and suggestions for increasing program adherence (such as suggestions about hours, the number of messages, and message frequency).

#### Procedure

Interviewers received training to (1) establish contact with potential participants, explain the study, and give them an informational brochure; (2) request consent for screening to identify inclusion criteria; (3) obtain informed consent from those who met the inclusion criteria and administer the initial evaluation questionnaire; (4) register recruited participants; and (5) inform participants that they could receive 100 pesos a month in cellphone credit to enable them to receive messages during the 4 months of the intervention and that they would receive the first messages no later than 2 weeks after registration. At the end of the intervention, participants were contacted again for a follow-up telephone interview. In recognition of their time and effort, those who participated in the follow-up interview were given a 200-peso gift card for groceries.

#### Data Analysis

Descriptive statistics were used for demographic variables, mental health indicators, and substance use. For the analysis of the open-ended questions, the meaning categorization technique was used, as explained in phase 1 of the study. The categories included are usability, acceptability, and adoption.

## Results

### Needs Assessment

#### Participant Characteristics

The sample included 21 migrants (14 men and 7 women), with an average age of 36 years (SD 10.47); 16 were in Morelia and surrounding locations, and 5 were in Tijuana. Half were married or living with a partner. The average time since returning to Mexico was 11 months (SD 8.97). Only one pair of interviewees had social security. The most common educational level was elementary or junior high school (Table 1).

**Table 1.** Characteristics of migrants and key informants.

Characteristics	Migrants (n=21), n (%)	Key informants (n=22), n (%)
Gender		
Men	14 (67)	9 (41)
Women	7 (33)	13 (59)
Marital status		
Single	9 (43)	13 (59)
Married	12 (57)	9 (41)
Educational level		
Elementary or junior high school	10 (48)	4 (18)
High school	7 (33)	2 (9)
Undergraduate	4 (19)	13 (59)
Graduate	0 (0)	3 (14)
Occupation		
Trade	12 (57)	—^a^
Employee	5 (24)	—
None	4 (19)	—
Health care	—	11 (50)
Organization manager	—	7 (32)
Government employee	—	4 (18)

a Not applicable.

There were also 22 key informants (13 women and 9 men) with an average age of 41 (SD 12.09) years. They had an average of 5 (SD 5.47) years of experience working with the migrant population. Most were single, and more than half had studied for a bachelor’s degree. They worked in government agencies that served migrants as well as in nonprofit organizations, shelters, and rehabilitation clinics for addiction, providing health care activities (psychology, medicine, and nutrition) or engaging in organizational coordination or management (Table 1).

#### Need for Services

Four noteworthy categories were identified. The first is related to the risk factors associated with emotional problems during return. These were grouped into 2 subcategories: family issues (eg, family separation or the loss of support) and social issues (eg, anger at deportation, difficulty in finding employment). The second included the resources and coping strategies that migrants used to manage their negative emotions; prominent among these were social support networks, self-care, healthy habits, and the reduction of risks associated with alcohol use. The third category consisted of barriers to seeking help, which were classified into personal (eg, beliefs and perceptions about mental health professionals) or structural (lack of resources or information). The fourth category referred to the viability of the psychoeducational strategy and included information about the accessibility of technology, its perceived usefulness, and suggestions (Table 2).

**Table 2.** Situational diagnosis: categories identified.

Categories and definitions, and subcategories		Examples
1. Risk factors: variables that favor or increase emotional problems during return		
Family risk factors		Family separation Loss of family support Conflict with partner or children “When not even my family wants to support me anymore, that’s when my world closes in.*”*
Social risk factors		Anger and frustration at deportation Difficulties in finding employment Social rejection and discrimination Limited access to housing and health services Alcohol use “At that moment [deportation], I felt like a failure; when your plans are not working out the way you expected, you start to feel frustration, which turns into anger and sadness.”
2. Resources and coping strategies: resources that migrants need or use to deal with their emotions and reduce their alcohol use		
Support networks		Support and communication with family and friends Share emotions with a trusted person “They give me strength. My wife and my children have told me: don’t fall into depression; you know you can always count on us.”
Self-care		Learn to manage emotions (anxiety, stress, anger, sadness) Develop abilities in communication, problem solving, and decision-making “To control myself a little, hold back a bit, because you know that anger—if you don’t control it—leads you to other things.”
Healthy habits		Involvement in activities including work, exercise, recreation, and leisure Reduce barriers to the adoption of balanced diet and sleep hygiene Promote participation in physical, recreational, and community activities “When I get angry, I’d rather leave the house and go for a walk to clear my mind. just walking and trying to change my thoughts so the sadness gradually goes away.”
Reduction in risks associated with use		Provide information about the risks of alcohol use Consider the impact of alcohol use on the family, health, and employment Strategies of risk and harm reduction Information about mental health services and alcohol use “Providing information on where to seek help, strengthening support networks. Not demonizing substance use or substances themselves. Addressing the underlying problem, not the consumption.”
3. Barriers to seeking help: obstacles to use of mental health services by migrants		
Personal		Belief in self-sufficiency and “willpower” Negative or erroneous perception of the work of mental health professionals Confusion about where to seek help (psychologist for emotions, physician for use) “We all think we go to a psychologist only when we’re already crazy, that’s why we don’t ask for help. I try to control myself on my own.”
Structural		Lack of economic resources or social security Lack of information about available services *Anexo* as a resource for dealing with alcohol use “[We do not use health services] due to lack of resources, because many times they are expensive and you don’t have insurance or anything like that.”
4. Viability of the psychoeducational strategy: perception of the usefulness of the message-based strategy and conditions for its implementation		
Technological accessibility		Most people have a smartphone Common use of WhatsApp Access to Wi-Fi and cellular data service (brief interruptions) SMS perceived as useful but not attractive “[WhatsApp] is the one we use the most, and you can receive images, videos, and audios.”
Perceived usefulness		Promotion of healthy habits and self-care Reflection on substance use Detection of emotional problems or problems of use Motivation to seek help Feelings of support, accompaniment, and creation of a social support network Limited efficacy for severe or long-term problems “For someone who is alone and depressed, receiving a message that provides emotional support can help them feel that they are not alone.*”*
Suggestions		Brief, visually attractive messages Positive, thoughtful language Varied formats: images, audio, video Weekly frequency (flexible) Two messages at a time Messages preferably in the evening (weekends for messages about use) “Messages should be short; if they are overwhelming, people won’t read them. They should include images, audios, and videos.”

### Content Development

**Figure 3.**
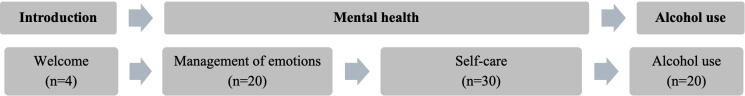
Sequence of topics and number of messages.

A total of 80 messages were created, of which 65% (n=52) focused on mental health (management of emotions and self-care), 25% (n=20) on alcohol use, and 10% (n=8) on the experience of migration. The latter were included at the beginning and the end of the intervention, and before each topic, to motivate participants to stay in the program. Figure 3 shows the sequence of the topics and the number of messages in each section.

**Figure 4.**
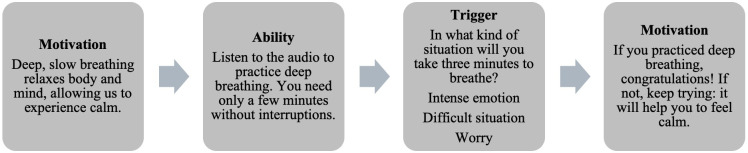
Example of the sequence of messages for approaching a topic.

Each topic was addressed with an average of 4 messages (motivation, ability, and triggers). The first message of the sequence offered information about the activity, highlighting the benefits of adopting the behavior; the second provided guidelines for carrying out the task; the third offered alternatives for facilitating involvement in the activity; and the fourth reinforced the success and the benefits of maintaining the change. Figure 4 shows an example of the sequence of messages.

**Figure 5.**
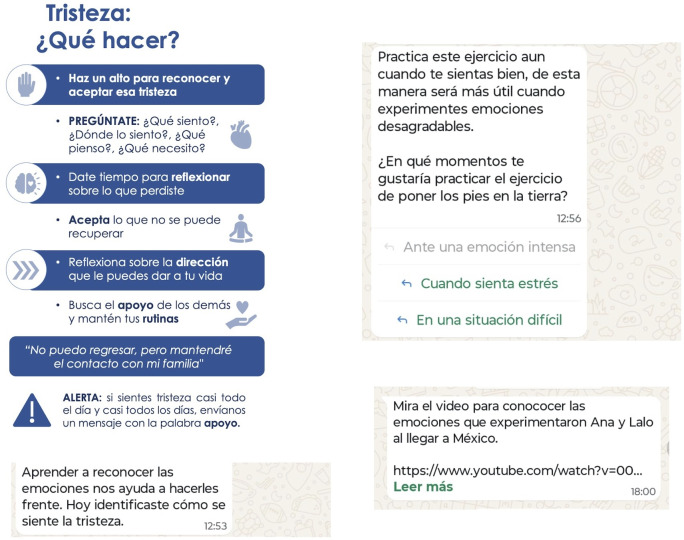
Examples of message formats.

The messages were created considering participants’ preferences for their characteristics and format. These coincide with recommendations in the literature [18] for increasing the effectiveness of SMS text messaging, including expression in positive terms, providing direction, and facilitating access to resources. The messages were brief and direct, using colloquial language, and with an emphasis on practical content. The information was presented in various formats: text, images, video, audio, and questionnaire (Figure 5).

### Expert Evaluation

The highest levels of agreement were observed regarding the appropriateness of the messages for the goals of the project, the number of messages in the program, the relevance of the messages to the topic, and their contribution to the section. Table 3 shows the average level of agreement in the evaluation criteria by question. Table 4 shows the averages by topic.

**Table 3.** Average evaluation score by question.

Evaluation criteria for each message	Introduction (n=7)	Experience of migration (n=7)	Management of emotions (n=2)	Self-care (n=2)	Alcohol use (n=3)
1. Relation to topic	4.8	4.8	5.0	4.9	5.0
2. Contribution to the section	4.7	4.8	4.9	4.9	4.8
3. Understandability of language	4.6	4.1	4.7	4.8	4.1
4. Relevance of information	4.7	4.6	4.9	4.9	4.6
5. Appropriateness of format	4.6	4.6	4.9	4.9	4.4

**Table 4.** Average evaluation score by topic.

Evaluation criteria for section	Management of emotions (n=2)	Self-care (n=2)	Alcohol use (n=3)
1. Appropriateness to purpose of project	5.0	5.0	5.0
2. Number of messages	5.0	5.0	5.0
3. Practicality of exercises	5.0	4.5	4.3
4. Appropriateness to target group	4.5	4.5	4.6
5. Motivational effect	5.0	4.5	4.6

The lowest scores were for the understandability of the language for some of the messages on the topics of experience of migration and alcohol use. The evaluators gave recommendations related to the precision of the information: for example, on the topic of alcohol use, to use the phrase “use that doesn’t put you at risk” instead of “to have lower risk with use.” Some evaluators identified the use of technical language and suggested more colloquial language, such as “headache” instead of “migraine” and “increased heartbeat” instead of “tachycardia.” They also suggested changes to enhance the motivational effect of the messages, such as using the phrase “when we allow ourselves to feel sadness” instead of “it is necessary to allow ourselves to feel sadness.”

The most common commentaries referred to the clarity, precision, conciseness, coherence, and practicality of the messages. Evaluators expressed their satisfaction with the psychoeducational strategy as a valuable option for creating change: “Concise, practical messages, with simple language. They generate interest, with tools that are easy to apply. They help each person to be responsible for their own changes; they succeed in providing accompaniment without being intrusive...”

Changes were made to some messages in response to the evaluators’ observations. No changes were made to the number of messages or their sequence. These changes resulted in a significant improvement in the content of the messages and the psychoeducational strategy.

The platform used to implement the program was BroadcasterBot, a messaging service that enables the scheduling and delivery of standardized message templates to predefined groups, in accordance with the Mexican Federal Law on the Protection of Personal Data Held by Private Parties [30], which regulates how companies handle personal data, guaranteeing privacy, and ARCO (Rights of Access, Rectification, Cancellation, as Well as Opposition to the Processing of Personal Data Held by Private Parties) rights. Communication was primarily unidirectional; however, participants were able to respond by selecting predefined response options. User inputs were monitored through the platform for research and statistical purposes. Tests were carried out among the research team, and once the proper functioning of the platform was confirmed, the pilot study was carried out.

### Pilot Study

A total of 14 migrants participated (9 women and 5 men; Table 5), aged 19 to 50 years (mean 30). The time since their return to Mexico ranged from a few days to 12 months (mean 5). Of the total, 11 completed the program.

**Table 5.** Characteristics of participants in the pilot study.

Characteristics	Men (n=5), n (%)	Women (n=9), n (%)	Total (n=14), n (%)
Marital status			
Single	4 (80)	7 (77.70)	11 (78.60)
Married	1 (20)	2 (22.30)	3 (21.40)
Educational level			
Elementary or junior high school	3 (60)	2 (64)	5 (35.70)
High school	2 (40)	2 (29)	4 (28.60)
College	0 (0)	5 (7)	5 (35.70)
PHQ-4^a^			
Normal	2 (40)	3 (33.30)	5 (35.70)
Mild	1 (20)	3 (33.30)	4 (28.60)
Moderate	1 (20)	2 (22.20)	3 (21.40)
Severe	1 (20)	1 (11.10)	2 (14.30)
AUDIT^b^			
Low	3 (85.70)	9 (100)	12 (85.70)
Risky	1 (7.10)	0 (0)	1 (7.10)
Harmful	1 (7.10)	0 (0)	1 (7.70)

a PHQ-4: 4-item Patient Health Questionnaire*.*

b AUDIT: Alcohol Use Disorders Identification Test.

Messages were programmed and sent to various groups, and no problems were encountered with the platform. The functions for personalization and monitoring participant interactions performed well.

Six participants were contacted for follow-up 3 months after the intervention. The results are summarized in Table 6.

**Table 6.** Follow-up results.

Category	Results
Usability	They were satisfied with the number and frequency of messages and the variety of formats used. The majority reported that they always or almost always read the messages and that they had no difficulties in responding.
Acceptability	They reported that the activities “are easy to understand and do not require much time to practice.” They would recommend the program to other migrants because “it helps you to feel better in both ways, physically and emotionally.” They showed a high degree of acceptance of the monitoring of their mood, which they saw as accompaniment.
Adoption	The majority put the recommendations into practice, mainly to maintain a healthy life and to deal with difficult emotions. “The information about changing your point of view and some of the activities about breathing really stuck with me. In general, they are the ones I still use the most when I have a problem or if I start to have a panic attack. It helps me to calm down and think about the situation more clearly.”

Although the interviewees did not suggest changes to the content or format of the messages, some changes were made to make them visually more attractive, and texts were synthesized to aid in their comprehension. The interviewers made suggestions for improving the evaluation questionnaire.

## Discussion

### Principal Findings

This study describes the process of creating a psychoeducational program to reduce the risk of mental health problems related to the experience of voluntary or involuntary return of a group of migrants. The development of the intervention includes various notable characteristics. First, both the identification of the most important topics and the development of content and the design of the distribution strategy were based on the needs expressed by a group of returning migrants. In this first stage, the major challenges identified by returning migrants included (1) the identification of emotional problems associated with the experience of migration, (2) the management of emotions and self-care, and (3) the use of psychoactive substances. These findings are consistent with those of other studies [4,31], which identify a variety of effects on mental health associated with the experience of migration.

A second important feature is the active collaboration of representatives of the target group and other actors in different stages of the process. This participatory methodology, also called coproduction, is an innovative way of finding responses or solutions to complex situations [32]. In this case, coproduction enriched the general aim of the project by incorporating the needs expressed by the migrants, the perspective of key informants, the production of videos, and the validation of experts on themes related to migration, mental health, and substance use. This process led to an appropriate psychoeducation program, based on research data and committed to the primary goal of improving the management of emotions and encouraging the development of habits of self-care, even in conditions of psychosocial vulnerability.

Coproduction also allowed for the identification of features of content and design that were or were not acceptable. The review by the target population at an early stage contributed to the development of relevant content, presented in comprehensible language that described situations consistent with the experiences of returning migrations, facilitating acceptance of the intervention [10]. The same was true for the technical aspects of the program: it was designed specifically for distribution using WhatsApp, the platform most used by the target population. It is easily accessible and, for many people, has no cost, which favors its adoption. The frequency and scheduling of messages were also determined by the coproducers. In future studies, it will be necessary to determine whether this characteristic contributed to adherence.

The use of coproduction allowed for the development of a culturally sensitive intervention for returning migrants, using a language and examples related to the needs of this population. In a study carried out with Syrian refugees, it was observed that this adaptation to the language, culture, and context of refugees improved the relevance, acceptability, and ease of use of the app [33].

A third important feature of this project is that, in addition to incorporating the points of view of different actors, the psychoeducational program included recommendations from international literature regarding the management of emotions and the modification of behavior using effective techniques that can be easily applied. This was corroborated in the pilot phase, where participants reported that it was easy to put the recommendations into practice and that they succeeded in recalling some of the messages. The pilot study found that 80% of the participants continued through the last of the 16 weeks of the program; this is an encouraging sign for the feasibility of the intervention, but a specific study will be required to assess adherence.

The intervention was found to be useful for helping participants to deal with daily emotional problems, with minimal synchronic intervention by a mental health specialist. Requests for support during the intervention consisted of a few interactions, one of which involved referral to a community service, which was also one of the messages of the program.

### Limitations

This study shows the different stages in the design of a psychoeducational program directed at returning migrants. Given that it is a study of process, the participants were not chosen to be statistically representative. However, this could constitute a limitation in generalizing the information about the experience of migration. Another limitation of the study is that the intervention can only be used by people who have a smartphone; it fails to reach those who are in even more vulnerable conditions.

This study outlines the different stages of designing a psychoeducational program for returning migrants. Participant selection focused on the depth and relevance of their migration experiences, rather than statistical representativeness. This selection procedure could limit the generalizability of the findings to other migration profiles. Another limitation is that the intervention is only accessible to individuals with smartphones, thus excluding those in even more vulnerable situations.

Furthermore, while the expert content evaluation phase followed a specialized design to ensure the content validity of the messages, a comprehensive psychometric evaluation of each message was not conducted. This analysis is a recommended step in developing digital interventions to strengthen their reliability. Although the 80% retention rate suggests the intervention’s viability, further research into the reasons for dropout among the remaining 20% was needed. Future research should incorporate a qualitative analysis to identify internal and external factors associated with dropout, thereby improving the intervention’s design and relevance.

### Next Steps

Future studies should evaluate, in the short and medium term, the effect of the psychoeducational program on emotional problems and the adoption of self-care habits. They should also identify the participant characteristics associated with greater adherence, the characteristics that predict attrition, and the best conditions for the implementation of the strategy.

### Conclusions

This study describes the different stages in the development of the psychoeducational program “Here Again: Coping With Return,” whose objective was to reduce the risks to mental health associated with a return to Mexico. The determination of topics to address, the language to be used, and the technological tools to distribute the content were the result of a process of coproduction, which is indispensable to the design of culturally relevant interventions. The use of coproduction allowed for an interchange of ideas, enabled diverse voices to be heard throughout the development of the intervention, and helped migrants to feel that they were part of the team and not just passive recipients.

## Abbreviations

ARCO: Rights of Access, Rectification, Cancellation, as Well as Opposition to the Processing of Personal Data Held by Private Parties
